# Barriers to perinatal healthcare for women with disabilities: A Narrative Review with a Systematic Search and Regional Focus on Kashmir^☆^

**DOI:** 10.1016/j.dialog.2026.100296

**Published:** 2026-03-18

**Authors:** Uroos Mahliqa, Zahid Ahmad Lone, Aadil Bashir, Sarafraz Ahmad

**Affiliations:** Department of Social Work, University of Kashmir, India

**Keywords:** Barriers, Healthcare, Kashmir, Perinatal period, Narrative review, Women with disabilities

## Abstract

Women with disabilities face significant barriers in accessing perinatal healthcare, often leading to poor maternal and neonatal outcomes. This narrative review, conducted using a PRISMA-informed systematic search strategy, aims to identify and synthesize the global literature on barriers to perinatal healthcare among women with disabilities, with a contextual sub-analysis of studies relevant to the Kashmir region.

A total of 61 studies, published between 1967 and 2024, were identified through a PRISMA-informed systematic search process across four academic databases: Psycinfo, Jstor, Medline, and Google Scholar. Studies were appraised using the Mixed Methods Appraisal Tool (MMAT). Given substantial heterogeneity in study designs, disability types, outcomes, and regional contexts, findings were synthesized using an interpretive narrative thematic approach rather than statistical aggregation. The review synthesized findings from both qualitative (*n* = 36) and quantitative (*n* = 25) studies, including 10 studies focused on Kashmir, highlighting a significant regional research gap.

Thematic synthesis identified three major themes: (1) barriers related to perinatal healthcare, (2) support systems during the perinatal period, and (3) healthcare system policy response and gaps. Barriers comprised physical inaccessibility, attitudinal stigma, knowledge and information barriers and socio-cultural barriers. Among the 61 studies reviewed, physical and attitudinal barriers were the most frequently reported (in 42 studies), followed by knowledge and information barriers, (in 28 studies) and socio-cultural barriers (in 25 studies). Support systems particularly emotional, familial and peer networks, were identified as important facilitators of perinatal healthcare access.

In the Kashmir context, these barriers are intensified due to political instability, under-resourced health infrastructure, and sociocultural stigma. These findings underscore the importance of disability-inclusive perinatal healthcare strategies and context-sensitive policy approaches.

## 1 Introduction

Perinatal period spans conception to one year after childbirth, emphasizing comprehensive care for the mother and the infant [1]. Care across these stages from preconception to postnatal support promotes healthy outcomes for both [2]. However, women with disabilities often face structural and systemic barriers in accessing adequate care and support [3]. Disabilities, whether physical, sensory, intellectual, or psychosocial, often intersect with socioeconomic marginalization, geographic isolation, and social stigma [4]. These intersecting factors compound the challenges women with disabilities face in accessing perinatal care [5], [6].

Inaccessible medical infrastructure, insufficient adaptive equipment, limited provider training, and discriminatory practices contribute to substandard care and adverse maternal health outcomes [5], [6].

Research from various contexts suggests that the intersection of gender and disability intensifies these barriers. Women with disabilities are often subject to marginalization and have limited recognition of their reproductive autonomy [7]. Healthcare systems often fail to accommodate their specific needs, resulting in inadequate prenatal monitoring, poor pain management during labour, and limited postnatal support [8]. Additionally, barriers to accessible health information constrain informed decision-making and active participation in their own care [9].

Although these issues are recognized globally, they are particularly pronounced in the region of Kashmir, where geographic, political, and cultural factors create a distinct context of vulnerability [10]. Recurrent conflict, mobility restrictions, and communication disruptions have consistently undermined healthcare delivery in Kashmir [11]. More than 40% of healthcare facilities in Kashmir lack basic obstetric equipment and skilled personnel [12]. These infrastructural gaps disproportionately affect women with disabilities and are further compounded by sociocultural stigma, which associates disability with familial shame. Such stigma contributes to underreporting of pregnancies and unsupervised home births, reported in 68% of cases [13].

*Prolonged political instability in Kashmir has led to infrastructural breakdowns,* limited healthcare access in remote areas, and chronic shortages of trained medical personnel [14]. Women with disabilities in Kashmir, already marginalized due to their impairments, face additional challenges such as restricted mobility, social isolation, and minimal provider awareness [15]. Traditional beliefs around disability and childbirth often discourage these women from seeking formal care, further compounding their exclusion within families and communities [16].

The limited availability of disability-inclusive reproductive health services and the lack of trained providers further deepen the disparities in maternal health outcomes in the region [17]. Despite efforts to improve maternal and child health in India, the perinatal experiences of women with disabilities in Kashmir remain largely undocumented and poorly understood. The convergence of disability, gender, and region-specific constraints makes it imperative to examine these experiences through a localized lens.

This study addresses this gap by investigating the perinatal challenges faced by women with disabilities in Kashmir. It aims to identify key barriers to accessing care, evaluate their impact on maternal and infant well-being, and explore the strategies these women use to navigate the healthcare system. It also seeks to understand how cultural norms and societal attitudes influence their experiences. By centering the needs of this marginalized population, the study contributes to broader efforts aimed at advancing inclusive healthcare and reproductive justice, particularly in underserved and developmentally constrained regions like Kashmir.

Promoting the reproductive rights of women with disabilities is a critical dimension of this research. These rights, anchored in principles of autonomy, equality, and dignity, are often undermined by systemic discrimination and a lack of accessible services. Ensuring equitable access to care and informed decision-making for women with disabilities is vital not only for individual well-being but also for achieving public health goals and sustainable development objectives.

Rather than being solely an individual condition, disability is understood as a socially produced, dynamic phenomenon shaped by environmental and institutional barriers that vary across social and cultural contexts [18]. In the context of Kashmir, understanding disability requires attention to local cultural narratives, historical neglect, and emerging efforts toward inclusion. By situating disability within both global frameworks and regional realities, the study highlights the need for disability-sensitive, gender-inclusive, and context-specific interventions to improve perinatal health outcomes for women with disabilities in Kashmir.

## 2 Methodology for the study

### 2.1 Review typology and rationale

This study was conducted as a *narrative review* with PRISMA- informed systematic search strategy. A structured and transparent search strategy was employed in accordance with PRISMA guidelines to identify relevant literature; however, due to heterogeneity in study designs, populations, disability types, and outcome measures, a meta-analysis was neither feasible nor appropriate. Instead, findings were synthesized using a narrative thematic approach, allowing for the integration of qualitative and quantitative evidence and for contextual interpretation, particularly with respect to the Kashmir region.

### 2.2 Search strategy

A comprehensive search was conducted across four major databases: Psycinfo, Jstor, Medline, and Google Scholar. A periodic database search was conducted between August 2024 and February 2025, targeting studies published over the last 50 years (1967–2024) to ensure relevance and timeliness. The inclusion criteria centred on research addressing barriers to perinatal healthcare faced by women with disabilities, globally, with a contextual sub-analysis of studies relevant to the Kashmir region.

**Fig. 1:**
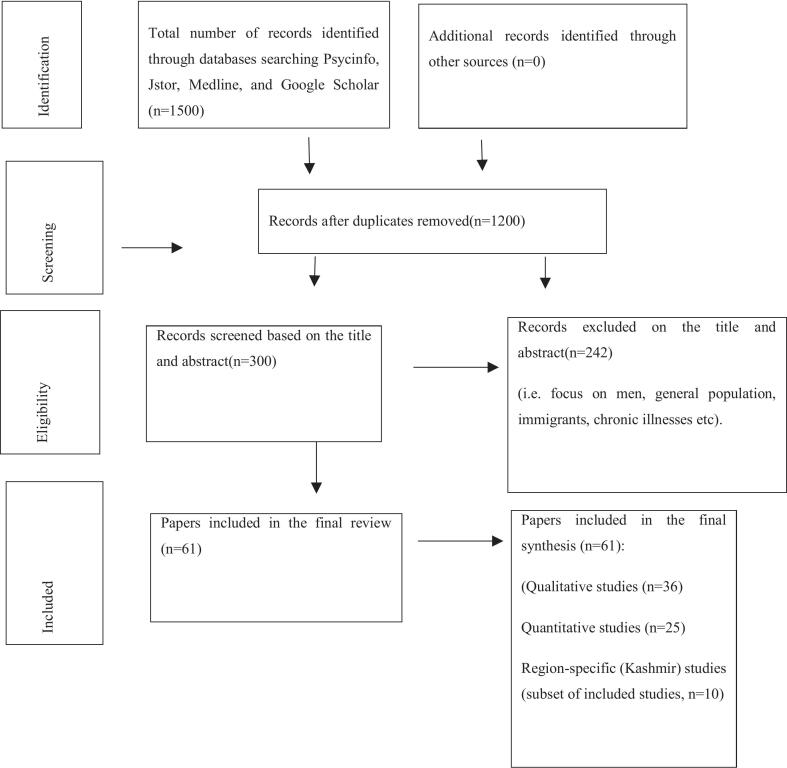
PRISMA Flow Diagram of the Literature Search and Study Selection Process.

Search terms included ‘perinatal period and disability’ ‘disabled women in Kashmir’ ‘reproductive health of disabled women’ ‘pregnancy complications among disabled women’ ‘maternal health challenges and disability in Kashmir’. The review followed a PRISMA-informed systematic search strategy. Reporting of the search and study selection process adhered to the Preferred Reporting Items for Systematic Reviews and Meta-Analyses(PRISMA) guidelines(Fig. 1) as outlined by Moher et al. [19]. However evidence synthesis was conducted using a narrative thematic approach due to the substantial heterogeneity across study designs populations and outcomes.

Boolean operators were used to refine the search strategy For example: (“perinatal period” AND “women with disabilities”), AND “Kashmir” which specified the results to relevant studies.

All the papers used in our study were exported to Mendeley software. Additional studies were identified from the reference list of the selected papers.

Grey literature, including government reports, conference abstracts, and unpublished thesis, was excluded to ensure that only peer-reviewed, full-text academic publications in English were analysed. Government reports, such as those from the U.S. Department of Justice, were referenced only for background context and were not included in the narrative synthesis dataset.

### 2.3 Eligibility criteria

The initial screening was based on titles and abstracts to determine the relevance of identified studies. Full-text articles were reviewed when abstracts lacked clarity regarding inclusion criteria. Each study's eligibility was assessed based on established criteria, emphasizing its relevance to the perinatal obstacles faced by women with disabilities.

### 2.4 Inclusion criteria

Studies were included if they: (1) focused on women with disabilities during the perinatal period, (2) examined barriers or challenges related to healthcare access, quality, or support systems, (3) employed qualitative, quantitative methods, or mixed-methods designs, and (4) were published in English between 1967 and 2024.

### 2.5 Exclusion criteria

Studies were excluded if they were unrelated to the perinatal health or disability among women. Studies without full-text access were excluded, as were those not published in the English language. In addition, studies published before 1967 were not considered for inclusion in the review.

### 2.6 Studies selection process

An initial search yielded (*n* = 1500) articles, of which (*n* = 1200) were excluded as irrelevant. Abstracts of the remaining articles (*n* = 300) were carefully reviewed, and full-text assessments were conducted for those with ambiguous abstracts. Out of the (n = 300) articles, (*n* = 242) were found to be irrelevant to the research topic. Ultimately, (*n* = 61) studies were selected, including 36 qualitative and 25 quantitative studies. Of these, 10 studies focused specifically on the Kashmir region. Although the reference list contains 64 citations, only 61 studies met the inclusion criteria and were included in the final synthesis; the remaining 3 references pertain to methodological frameworks such as PRISMA guidelines, the MMAT tool, and Braun and Clark's thematic analysis approach and were not a part of the review dataset.

### 2.7 Data retrieval and analysis

A structured approach was employed to gather information from the selected studies, focusing on elements such as: authorship, publication date, geographic context, study design and key findings. This involved a structured process of reviewing and categorizing the material to ensure accuracy and consistency. Data were extracted independently by two reviewers, and discrepancies were resolved through discussion and consensus.

For the qualitative component, manual coding was employed following Braun and Clarke's (2019) six phase framework for thematic analysis- familiarization with data, generation of initial codes, searching for themes, reviewing themes, defining and naming themes, and producing the final synthesis. This approach enabled systematic identification of patterns across included qualitative studies [20].

Qualitative findings were synthesized thematically, while quantitative findings were summarized descriptively to identify patterns and trends.

The synthesis identified three primary themes including: (1) barriers to perinatal healthcare for women with disabilities, (2) support systems for women with disabilities during the perinatal period, (3) and healthcare system policy response and gaps.

All stages of screening and data extraction were conducted independently by two reviewers, and disagreements were resolved through discussion and consensus.

### 2.8 Quality appraisal

To ensure methodological rigor and minimize risk of bias, a quality appraisal of all included studies was conducted using the Mixed methods appraisal tool (MMAT) version 2018 [21]. This tool is specifically designed to appraise the quality of qualitative, quantitative, and mixed methods studies within reviews that include divergent study designs. The assessment was carried out by two reviewers, and disagreements were resolved through discussion and consensus. The inclusion of a critical appraisal is aligned with the PRISMA (Preferred Reporting Items for Systematic Reviews and Meta-Analyses) guidelines which recommend systematic assessment of included studies for quality and bias. The PRISMA flow diagram is presented to enhance transparency of the search and screening process and should not be interpreted as indicating a meta-analytic or effect-based systematic review. PRISMA was used to ensure transparent and reproducible reporting of the search, screening, and selection process, while evidence synthesis followed a narrative thematic approach.

Each study was assigned an overall quality rating of high, medium, or low based on a general appraisal of its methodological soundness in accordance with MMAT domains. The appraisal focused on clarity of research questions, appropriateness of methodology, coherence between data collection and interpretation, and transparency of findings. Relevance to the study's aim-barriers to perinatal healthcare for women with disabilities- was also evaluated. All studies were retained in synthesis, but quality and relevance ratings were considered in the interpretation of findings.

A summary of the MMAT-based appraisal, including study design, overall quality rating and relevance to the review objectives, and brief contextual notes is provided in Table 1 (Supplementary Material). In Table 1, “Author(s) and Year” identify the source, “Study Design” indicates the methodological approach, “Quality” reflects the overall MMAT-based appraisal (high/medium/low), “Relevance” denotes alignment with the review objectives, and “Notes” provide brief contextual remarks. This table allows readers to quickly assess the methodological rigor and focus of each study. The MMAT ratings are intended to contextualize methodological rigor and should not be interpreted as a basis for ranking or excluding studies.

## 3 Ethical considerations

As this study involved a synthesis of previously published research and did not involve direct interaction with human participants or the collection of personal data, Institutional Ethical Review Board (IRB) approval was not required.

## 4 Results

Drawing on thematic analysis of 61 studies, we have structured the findings into three major themes: barriers to perinatal healthcare, support systems during the perinatal period, and healthcare system policy response and gaps. Table 2 provides key insights for each theme, along with their sub-themes. (See Table 3.)

**Table 2:** Key Themes and Sub-Themes on Perinatal Healthcare for Women with Disabilities.

Themes	Sub-themes	Key Insights
1.Barriers to Perinatal healthcare for Women with Disabilities	a). Physical barriers b). Attitudinal Barriers and Stigma c). Knowledge and Information Barriers d). Social and cultural barriers	Lack of accessible infrastructure, specialized equipment leads to poor maternal care and higher risks of complications like premature birth and low birth weight [21], [23], [24]. Prejudiced views from healthcare professionals question the reproductive roles of women with disabilities. Cultural beliefs in Kashmir further deepen this exclusion [25], [26], [27]. Lack of disability specific information; poor provider familiarity, weak communication; limited awareness of reproductive rights [9], [28], [29], [30], [31]. Societal beliefs about motherhood capability; stigma within families and communities' cultural myths about disabilities [24], [32], [33].
2.Support systems for Women with disabilities during the Perinatal Period	a). Peer support groups b). Familial Support c). Formal and Informal Support	Peer support offers emotional and informational support, promote sharing of coping strategies, and boost self-confidence and decision making [35]. Critical for managing pregnancy stress and providing emotional/practical help. Some families are supportive, while others maybe unsupportive or doubtful [36], [37], [38], [39]. Formal (healthcare) and informal(family, friends) support ensure access to care and support. Collaborative services sensitive to disability-related concerns are essential [40], [42], [43], [44].
3. Healthcare system policy response and gaps	a). Policy deficiencies b). Inadequate training of providers c). Systemic and infrastructure challenges d). Recommendations	Existing policies do not fully address the specific needs of disabled perinatal women, especially in Kashmir's unique setting [48], [54]. Healthcare workers ls often lack training to address disability-specific needs, resulting in poor communication and inadequate training [6], [49], [50], [51], [52]. Geographic challenges, poor transportation and lack of disability-inclusive health planning [51], [52], [54]. Inclusive policies, specialized perinatal care guidelines, culturally competent training, accessible infrastructure are needed [46], [48], [49], [53].

Note: This table summarizes themes extracted from 61 studies included in the systematic review. Sub-themes were derived using narrative synthesis. Some themes may overlap across categories.

**Table 3:** Descriptive overview of reviewed studies (n = 64) including authors, year, study design and key focus area.

Study No	Author(s)	Year	Country/Region	Design	Key Focus Area
1	Dean SV et al.	2014	Global	Narrative Review	Overview of maternal care continuum
2	Hill B et al.	2020	Global	Network Paper	Maternal obesity and global policy priorities
3	Blair A et al.	2022	Global	Scoping Review	Maternity care for women with disabilities
4	Jackson-Best F et al.	2018	Global	Systematic Review	Stigma and intersectionality in maternal care
5	Nguyen TV	2021	Vietnam	Qualitative	Lived experience of women with disabilities
6	Gleason JL et al.	2021	USA	Quantitative Cohort	Maternal risks in disabled women
7	Ngwena CG	2018	International Law (CRPD)	Legal Analysis	Reproductive autonomy and CRPD framework
8	McCauley H et al.	2022	Global	Systematic Review	Postnatal care in disability context
9	Hughes RB et al.	2022	USA	Qualitative	Health information seeking by disabled women
10	Ganguly S & Bajpai K	1994	India (Kashmir)	Political Analysis	Kashmir conflict; low relevance to health/disability
11	Ilyas M	2024	India (Kashmir)	Social Science	Peace education; indirect link to disability
12	Khanday SA	2024	India (Kashmir)	Review	Public health in Kashmir
13	Farooq T & Manzoor S	2022	India (Kashmir)	Qualitative	Parenting experiences of disabled in Kashmir
14	Khan HN & Digal G	2023	India (Kashmir Border)	Mixed Methods	Women's health issues in border regions
15	Ahmad S et al.	2024	India (Kashmir)	Qualitative	Social stigma and derogatory labels
16	Begley C et al.	2009	Global	Qualitative Review	Barriers to maternity care for disabled women
17	Wani RT et al.	2019	India (Kashmir)	Cross-sectional	Family planning knowledge among healthcare workers
18	Farooqi N & Ali M	2023	Global	Literature Review	Disability paradigms overview
19	Moher D et al.	2009	Global	Methodology Paper	PRISMA guidelines for systematic reviews
20	Braun V & Clarke V	2019	Global	Methodological Paper	Reflexive thematic analysis
21	Hong QN et al.	2018	Global	Methodological Tool	Mixed Methods Appraisal Tool (MMAT) for quality appraisal
22	Sharma R et al.	2023	India	Mixed Methods	Reproductive services accessibility for disabled
23	Deierlein AL et al.	2021	USA	Systematic Review	Pregnancy outcomes in disabled women
24	Tak JA	2022	India (Kashmir)	Journalistic Article	Lived realities of disabled women in Kashmir
25	Pathak M	2022	India (Kashmir)	Journalistic Article	Disability and healthcare system neglect in Kashmir
26	Addlakha R et al.	2017	Global (Theoretical)	Review	Sexual and reproductive rights of disabled women
27	Becker H et al.	1997	USA	Qualitative Study	Reproductive healthcare of women with disabilities
28	Bashir N & Dar JA	2023	India (J&K)	Review	Maternal health challenges for disabled women
29	Matin BK et al.	2021	Global	Systematic Review	Barriers to healthcare access for women with disabilities
30	O'Connor-Terry C & Harris J	2022	USA	Qualitative	Pregnancy decision-making by disabled women
31	Hajira S	2023	India	Policy Review	Disability rights policy in India
32	Farooq A et al.	–	India (Kashmir)	Report/Review	Public health system challenges in Kashmir
33	Nguyen TV et al.	2019	LMICs	Qualitative Review	Maternal care for disabled women in low- and middle-income countries
34	Smith E et al.	2004	Zambia	Qualitative	Barriers to reproductive care for disabled women
35	Potvin LA et al.	2016	Canada	Qualitative	Social support for women with intellectual disabilities
36	Lawler D et al.	2013	International	Systematic Review	Access to maternity care for disabled women
37	Jamieson R et al.	2016	UK	Qualitative	Supported decision-making for mothers with disabilities
38	Payne DA et al.	2014	New Zealand	Qualitative	Maternity care during pregnancy and birth for disabled mothers
39	Carvalho CFS & Brito RS	2016	Brazil	Qualitative	Support networks in pregnancy for disabled women
40	Powell RM et al.	2017	USA	Qualitative	Family attitudes toward disabled women's pregnancies
41	Holmes TH & Rahe RH	1967	USA	Psychometric Scale	Stress scale — indirect relevance to maternity/disability
42	Höglund B & Larsson M	2013	Sweden	Qualitative	Motherhood experiences of intellectually disabled women
43	Lantz PM et al.	2005	USA	Survey	Role of doulas in maternal care
44	Tarasoff LA	2015	Canada	Literature Review	Perinatal experiences of women with physical disabilities
45	Guerin BM et al.	2017	New Zealand	Qualitative	Recommendations to improve maternity support for disabled women
46	Mitra M et al.	2016	USA	Mixed Methods	Unmet needs of pregnant women with disabilities
47	Shikako K et al.	2023	Global (14 countries)	Policy Analysis	Government disability responses during COVID-19
48	US Dept. of Justice	2020	USA	Policy Guidance	Medical care access for mobility-disabled persons
49	Bhat FA et al.	2014	India (J&K)	Cross-sectional	Gender and healthcare service access
50	Smeltzer SC et al.	2016	USA	Qualitative	Perinatal care experiences of disabled women
51	Casebolt MT et al.	2023	India (Rajasthan)	Secondary Data	Maternal healthcare utilization among disabled women
52	Mitra M et al.	2017	USA	Qualitative	Healthcare provider perspectives on disabled maternity care
53	Homeyard C et al.	2016	UK	Systematic Review	Antenatal care for women with intellectual disabilities
54	Saeed G et al.	2022	Canada	Qualitative	Communication barriers in perinatal care
55	Mason MG	2012	USA	Book	Insights from mothers with disabilities
56	Zahoor N et al.	2020	India (Kashmir)	Qualitative	Tribal pregnant women's health-seeking behavior
57	Fritsch K	2017	USA	Book Chapter	Disabled parenting and neoliberalism
58	Kayama M et al.	2021	India	Qualitative	Disability and social stigma in Indian context
59	Ven C et al.	2025	Global	Scoping Review	Factors influencing disability-inclusive maternity care
60	Smith DD & Tyler NC	2011	International	Review	Inclusive education less direct to health/disability
61	Sukhera J	2019	USA	Review	Empathy training in healthcare
62	Pinto-Coelho L et al.	2023	Portugal	Experimental Study	VR tools for improving empathy in perinatal care
63	Tarasoff LA	2017	Canada	Qualitative Review	Barriers to perinatal care for women with physical disabilities.
64	Piepzna-Samarasinha LL	2018	USA/Canada	Book	Disability justice, collective care, intersectionality, and community access

### 4.1 Barriers to perinatal healthcare for women with disabilities

Perinatal healthcare access for women with disabilities is hindered by significant obstacles, which leads to poor mother and new-born health outcomes. In the context of Kashmir, these obstacles are multifaceted and result from systemic difficulties like physical inaccessibility, insufficient medical infrastructure and widespread societal stigma resulting in a challenging landscape for disabled perinatal women.

#### 4.1.1 Physical barriers

Physical inaccessibility to healthcare services creates significant barriers for women with disabilities. Many hospitals and clinics lack essential infrastructure to accommodate people with mobility limitations, negatively impacting the quality of healthcare services available to them especially during the perinatal period. Basic amenities such as ramps, accessible examination tables and specialized equipment for gynaecological procedures are often missing. The literature repeatedly highlights that these physical barriers prevent women with disabilities from receiving timely and effective care, increasing their risk of complications during the perinatal period [22]. Women with disabilities face increased vulnerability to adverse perinatal outcomes. These include higher risks of preterm labour and low birth weight, both of which can lead to serious maternal and neonatal complications. Despite known concerns, limited research on maternal health issues among women with disabilities has created gaps in understanding these challenges and contributing factors [23]. In regions like Kashmir, inaccessible public buildings, transportation, and educational institutions exacerbate these challenges, fostering dependency and restricting access to essential healthcare and daily activities, thereby limiting women's ability to receive timely and appropriate care [24], [25].

In Kashmir, inaccessible public buildings, transportation, and educational institutions exacerbate these issues, fostering dependency and restricting access to essential healthcare and daily activities [24], [25].

#### 4.1.2 Attitudinal barriers and stigma

Societal attitudes toward women with disabilities pose significant barriers to accessing perinatal healthcare. Many healthcare providers lack the awareness and sensitivity required to address their specific maternal health needs. As a result, these needs are often met with limited empathy and understanding, further compounding the challenges that they face. Such interactions have been reported to contribute to fear and mistrust among women with disabilities. According to studies, medical personnel may hold prejudices, often perceiving women with disabilities as unfit to be mothers [26]. Such stigmatized attitudes can limit adequate support, leaving these women without proper guidance during the perinatal period. As a result, these women's reproductive needs may be disregarded. Improper counselling, neglect and inadequate prenatal and postnatal care are some of the ways in which prejudice may appear. Traditional cultural beliefs in Kashmir significantly influence healthcare practices and intensify stigma toward women with disabilities [27]. This stigma can impede autonomy and delay care-seeking [28].

#### 4.1.3 Knowledge and information barriers

Disabled perinatal women frequently experience significant barriers in accessing accurate and relevant health information related to their perinatal period. Many face healthcare practitioners lacking experience with disability-related needs, which can lead to them feeling misunderstood, unsupported and vulnerable throughout their perinatal journey. In addition, disabled perinatal women often face attitudinal barriers from healthcare practitioners, who may hold biases or preconceived notions about their ability to bear children. Some describe being discouraged from becoming pregnant or experiencing judgemental behavior from medical staff. Many healthcare systems fail to provide clear disability-specific guidance on delivery options, family planning and maternal healthcare [9], [29].

Without essential resources and information women with disabilities struggle to manage their maternal needs and make informed decisions. Many remain unaware of their options and available practices due to insufficient specialized maternal wellness advice [30].

In Kashmir, these challenges are intensified by limited health education outreach, poor internet access in remote areas, and a lack of disability-inclusive awareness. Community health workers often lack training to communicate effectively with women with disabilities, especially regarding sensitive issues like pregnancy and reproductive health [31]. Consequently, many remain uninformed about their reproductive rights, perinatal care, and support services, leaving them isolated during this crucial period [32].

#### 4.1.4 Social and cultural barriers

Social and cultural barriers significantly shape the experiences of women with disabilities during the perinatal period. Societal beliefs often regard women with disabilities as asexual, leading to widespread doubts about their sexuality and parenting abilities. They are often perceived as incapable of being good mothers and their decision to have children is viewed as a burden, both to themselves and for their families overshadowing the joy and fulfilment that motherhood may provide to these women [33].

In Kashmir, disabled persons continue to face societal marginalization and stigma, resulting in a lack of acceptance and support from both families and communities. This exclusion hinders their integration into the mainstream society [25]. Numerous social and cultural challenges hinder women with disabilities from accessing maternal wellness care services. Social stigma surrounding disabilities often perpetuates misconceptions and beliefs that adversely affect women's health and autonomy. Cultural myths, such as the belief that disabilities are hereditary or that women with disabilities are unfit for motherhood, further marginalize them. These ingrained views can lead families, communities, and even medical professionals to doubt the legitimacy of their reproductive choices [34].

### 4.2 Support systems for women with disability during the perinatal period

Emotional and social assistance frameworks are crucial for perinatal women with disabilities, who often experience heightened social isolation, stress, and stigma. Physical limitations can intensify these challenges, while cultural attitudes that devalue their maternal experiences further contribute to emotional distress [35]. Addressing these challenges requires a coordinated system of peer, familial, and professional support that collectively enhances women's confidence, resilience, and ability to navigate the perinatal period [36]. Taken together, these findings demonstrate that support systems are not supplementary, but central to navigating perinatal care for women with disabilities.

Peer support groups tailored to mothers with disabilities offer a safe space for women to share their experiences, challenges, and coping strategies. These groups play a central role in fostering solidarity and empowerment. They offer emotional reassurance, as well as practical guidance on adaptive parenting, accessible birth options, and self-advocacy in healthcare settings [37]. Peer groups and community-based networks help bridge this gap by providing spaces where women can share lived experiences, coping strategies, and self-advocacy skills within supportive and non-judgmental environments. Participation in such peer engagement has been shown to enhance confidence, promote informed decision-making, reduce anxiety, and increase women's preparedness for childbirth [38].

Alongside peer networks, family and intimate relationships form the most immediate and influential layer of care, providing both emotional and practical support. Women with disabilities during perinatal period may rely largely on family members or caregivers to assist them with daily activities, mobility, and parenting obligations. This support fosters an inclusive and supportive environment for the mother, enabling her to focus on her own well-being and the care of her child [39].

When families respond with acceptance and encouragement, they foster an inclusive and nurturing environment that enables women to focus on recovery and infant care. However, familial responses are not always positive; some families express uncertainty regarding the mother's health, reproductive decisions or ability to raise a child [39]. Such ambivalence reflects broader socio-cultural attitudes that shape the quality of familial support. Households facing social or economic pressures may offer limited assistance, whereas those grounded in empathy and understanding tend to enhance the mother's confidence, sense of autonomy and overall adjustment to motherhood [40].

Social support plays a crucial role in shielding women from the adverse effects of emotional distress and anxiety [41]. Formal and informal institutional assistance, including healthcare professionals, counselors, and community organizations, complements peer and family networks. Formal support, such as professional health services, counselling, and community programs assists with essential responsibilities like planning, transportation, and advocacy. Trained specialists familiar with disability-related concerns help women manage medical appointments, access information and make informed decisions about childbirth and parenting [42]. For instance, midwives and healthcare providers with disability-inclusive training have been shown to improve communication, empathy, and comfort during labour [43].

Informal support from family, friends, neighbours, or caregivers provides practical aid with daily duties and emotional encouragement, filling gaps where formal systems fall short. In addition, semi-formal companions such as doulas can offer continuous emotional and informational support during labour and postpartum. Together, these informal and semi-formal supports often accompany women to medical visits, assist with household duties, reduce stress, and enhance preparation for childbirth [44].

This multi-layered network of peer, familial, formal, and informal support is especially vital for women with disabilities, who are often considered a high-risk group in maternal care. Research shows that consistent social and informational support significantly reduces emotional stress and supports smoother transitions into motherhood [35], [42], [44]. When tailored to specific needs, support networks empower women to make informed decisions during pregnancy and manage motherhood responsibilities more effectively [45]. Conversely, inadequate emotional or practical support can have serious repercussions on perinatal health, leaving women anxious, overwhelmed, and less equipped to care for themselves and their new-borns [38].

### 4.3 Healthcare system policy response and gaps

Despite growing global concern, research on the unmet perinatal needs of women with physical disabilities remains limited. Studies identify several key gaps, including inadequate clinical expertise, negative provider attitudes, inaccessible infrastructure, and a lack of disability-specific perinatal care information. Many providers fail to address disability-related concerns, and healthcare facilities often lack accessible equipment [46].

At policy level, many countries have implemented inclusive health policies emphasizing non-discrimination, informed consent, and infrastructure adaptations such as adjustable examination tables and accessible birthing suites- comprehensive provider training remains crucial to ensure equitable maternal care [47], [48].

Regionally, in Jammu and Kashmir, healthcare improvements remain insufficient due to poor road connectivity, scattered communities, harsh weather conditions, limited private sector involvement, and inadequate coordination between state and central governments, especially regarding women's healthcare [49]. A qualitative study by [50] with women with physical disabilities highlighted three key challenges: clinicians' limited awareness of disability-specific pregnancy issues, disregard for women's lived experiences, and a lack of recognition of disability-related maternal risks. Participants emphasized the need for improved perinatal care, including addressing problematic provider interactions and implementing more tailored care strategies.

Complementing these regional insights, Casebolt et al. [51] examined the relationship between disability and maternity healthcare utilization among married women in Rajasthan, India, using data from the first wave of the Indian Annual Health Survey (2011). Their analysis, which included 141,983 women aged 15–49 who had given birth between 2007 and 2009 highlighted persistent disparities in access to maternal healthcare services for women with disabilities. These findings emphasized the need for targeted interventions to improve both access and quality for this population.

A critical challenge in advancing disability inclusive maternal health care is the lack of disability-specific clinical data to inform policy and practice. This is compounded by four interrelated categories of barriers: evidence-related gaps, practitioner level hesitance stemming from inadequate training clinical practice limitations and system level barriers such as time constraints and restrictive reimbursement policies [52].

Providing adequate care requires adapting perinatal services including antenatal care, communication, and health information to the specific needs of women with disabled. Many midwives report needing additional guidance due to limited training in disability-inclusive care [53]. Communication is another major barrier, particularly for those with sensory or intellectual disabilities, as providers often lack experience, training, and supportive regulatory frameworks. Research shows that women with disabilities face substantial barriers to communication when seeking perinatal care. Studies reveal that women with sensory impairments and intellectual disabilities (IDD) face severe communication barriers during perinatal care. These impediments include the absence of necessary regulations and guidelines, insufficient provider experience, a lack of effort by healthcare personnel, and assumptions made by providers [42], [53].

In response, maternal and child health programs can improve outcomes by integrating disability-inclusive training and ensuring better representation of women with disabilities. This would close current gaps and promote equitable healthcare access [54].

In regions like, Jammu and Kashmir, challenges persist due to poor infrastructure, harsh weather, and lack of specialized staff and inclusive planning. Limited outreach, inaccessible transport, and undertrained providers continue to obstruct maternal healthcare for women with disabilities. Additionally, the absence of disability considerations in public health planning and limited outreach efforts highlight the need for inclusive policies and provider education tailored to regional challenges.

## 5 Discussion

As a narrative review conducted using a PRISMA-informed systematic search strategy, this study does not claim exhaustive coverage or statistical generalizability. Instead, its contribution lies in synthesizing diverse empirical findings through a disability justice and regional lens, allowing for deeper contextual interpretation of how structural, sociocultural, and political forces shape perinatal healthcare access for women with disabilities, particularly in politically instable settings such as Kashmir.

The findings of this paper reveal that women with disabilities face intersecting barriers to perinatal care, shaped by a complex interplay of structural neglect, cultural stigma, and systemic inequities. While these challenges mirror global patterns of healthcare exclusion such as inaccessible infrastructure and provider biases [3], [5] the sociopolitical context of Kashmir intensifies their severity in ways rarely observed in more stable or resource-rich settings. For instance, studies from high-income countries often attribute gaps in care to bureaucratic inefficiencies, such as rigid insurance policies or fragmented care coordination [46]. In contrast, Kashmir's prolonged political instability has led to disruptions in healthcare service delivery: healthcare facilities in remote areas are often inaccessible due to damaged infrastructure or unpredictable mobility restrictions. These context-specific constraints intensify existing healthcare inequities and limit the effectiveness of conventional maternal health interventions in Kashmir [12], [32].

Understanding these gaps requires more than technical fixes; it requires a framework that situates healthcare barriers within broader structural and social inequalities. This paper draws on the Disability Justice framework advanced by Piepzna-Samarsinha (2018), which positions care in relation to structural inequalities [55].

This framework helps analyze perinatal exclusion as more than a technical issue especially in regions like Kashmir where healthcare systems and social norms limit access for women with disabilities [56]. Applying this lens reveals that perinatal exclusion is not a neutral systems failure. Rather, it is the outcome of historically entrenched power hierarchies that rendered certain bodies- particularly women with disabilities – less visible to policy and healthcare design.

Stigmatising cultural narratives- that frame disability as familial shame or divine punishment [25] lead to pregnancy concealment and home births aimed at avoiding scrutiny [24]. These patterns raise critical questions about the relevance of global maternal health strategies such as digital outreach and centralized care coordination in regions facing infrastructure and sociopolitical constraints: Can such models function when institutional support is fragmented or non-existent? [54].

A closer examination of the literature also reveals divergent patterns within the Kashmiri context. While some families offer emotional and practical support, others exercise coercive practices, including discouragement from childbearing or enforced sterilizations [7], [32], [39]. These divergent responses may reflect socio-economic stratification, where families under greater financial strain are less likely to perceive women with disabilities as capable or independent [48]. However, regional studies often fail to disaggregate different types of disabilities, overlooking how experiences vary between physical, sensory, and intellectual impairments [18], [29], [53]. For instance, navigating rugged terrain poses different challenges for wheelchair users compared to communication barriers faced by women with sensory impairments [51]. Such homogenization reduces the utility of findings and highlights the need for intersectional research that accounts for factors such as disability type, socio-economic status, and rural-urban location [4].

The predominance of qualitative studies within the reviewed literature adds richness but limits generalizability. Quantitative data on adverse outcomes such as postpartum depression or neonatal complications remain scarce, and the near absence of longitudinal data makes it difficult to assess how changes in policy or sociopolitical stability impact maternal outcomes over time [23], [29].

Policy-level gaps equally persist. While national legislation such as the Rights of Persons with Disabilities Act (2016) mandates inclusive healthcare [31]. Its implementation in Kashmir remains limited, reflecting the systemic under-prioritization of disability inclusion in regional policy and resource allocation [48]. These deficits are visible at the ground level: maternity clinics in the region lack basic equipment like adjustable examination tables [21]; healthcare professionals often lack training in disability-competent care [49]; and community health workers rarely receive instruction in communication methods such as sign language [54]. These gaps are not isolated oversights but structural shortcomings that reinforce exclusionary practices. Without addressing the root causes of marginalization, including socioeconomic inequality, sociocultural stigma, and political instability, well-meaning policies and awareness campaigns may remain ineffective or even deepen disparities [10], [23].

Addressing these challenges requires a paradigm shift that prioritizes participatory frameworks centered on the lived experiences of disabled women. Globally recognized models such as trauma-informed care and peer support networks [37] must be adapted to Kashmir's sociopolitical landscape, considering variables like geographical isolation and unreliable digital infrastructure [57]. Locally tailored interventions such as mobile clinics or disability-inclusive home visits could provide more reliable access to care in hard-to-reach areas [51]. Training programs must not only focus on disability accommodation but also foster empathetic communication and cultural sensitivity among healthcare providers [58], [59]. These efforts should be paired with increased health budget allocations and integration of disability-specific indicators into maternal health monitoring frameworks [32], [46].

Thus, enhancing perinatal care for women with disabilities in Kashmir is not merely a technical challenge but a public health and ethical priority. It demands systems-level changes that reaffirm reproductive autonomy as a fundamental right [7] and create healthcare environments that support rather than hinder women with disabilities. As the disability justice framework insists, efforts to advance equitable perinatal care must address structural and systemic barriers that perpetuate exclusion.

Bridging the gap between global health paradigms and the lived realities of women in politically unstable settings is essential for realizing reproductive justice and upholding human dignity in all circumstances [32].

## 6 Limitations

To contextualize the findings, several limitations of this narrative review conductd using a PRISMA-informed systematic search strategy must be acknowledged. The focus on Kashmir, with 10 out of 61 studies being region-specific may limit the generalizability of the results to other regions with different cultural, social, and healthcare contexts. Despite analyzing 61 studies, the existing literature on this topic remains relatively sparse, and may not fully capture the complexities faced by perinatal women with disabilities.

Additionally, most Kashmir-specific studies focused primarily on physical disabilities, with limited exploration of sensory, intellectual, or psychosocial disabilities. This lack of diversity restricts a comprehensive understanding of how disability type influences perinatal experiences and healthcare access in the region.

The predominance of qualitative evidence limits the ability to draw statistically generalizable conclusions. While the review offers valuable insights into the key barriers faced by women with disabilities, it highlights the need for further primary research directly involving the target population, including longitudinal and quantitative studies, as well as the development of inclusive, context-specific interventions.

Furthermore, the exclusion of grey literature, non- English studies may have resulted in the omission of relevant regional evidence, potentially influencing the comprehensiveness of the synthesis.

## 7 Conclusion

Despite these limitations, the review highlights the need for a comprehensive and context-specific approach that addresses both structural and non-structural barriers. Inaccessible healthcare facilities and a lack of specialized equipment frequently result in inadequate perinatal care. In addition, societal and healthcare professional biases continue to limit opportunities for women with disabilities to receive competent care.

Targeted training programs that promote empathy, inclusion and disability-sensitive healthcare practices are essential to addressing these challenges [60], [61]. Such training enables practitioners to provide care responsive to individual needs thereby enhancing patient- provider interactions and health outcomes.

Cultural and societal stigmatization further discourage women with disabilities from seeking perinatal care [62]. Incorporating disability-sensitive counselling and educational programs into the healthcare system could create a supportive environment where women with disabilities feel empowered to make informed decisions about their perinatal health [63], [64].

Inclusive policies, accessible facilities, and trained disability-competent providers are critical for improving perinatal outcomes for women with disabilities. In Kashmir, participatory approaches centring women's voices may help align practice and policy. Combining context-specific research with inclusive policy can help build a rights-based healthcare model that improves the perinatal outcomes while affirming the autonomy and dignity of all women with disabilities.

## CRediT authorship contribution statement

**Uroos Mahliqa:** Writing – review & editing, Writing – original draft, Conceptualization. **Zahid Ahmad Lone:** Writing – original draft, Methodology, Investigation. **Aadil Bashir:** Supervision, Conceptualization. **Sarafraz Ahmad:** Supervision, Conceptualization.

## Funding

This research did not receive any specific grant from funding agencies in the public, commercial, or not-for-profit sectors.

## Declaration of competing interest

The authors declare that there is no conflict of interest.

## Appendix A Supplementary data

Supplementary material MMAT table Image 1
