# Supplementary material for: Barriers to perinatal healthcare for women with disabilities: A Narrative Review with a Systematic Search and Regional Focus on Kashmir
Source: Dialogues Health. 2026 Mar 18;8:100296. doi: 10.1016/j.dialog.2026.100296 (PMC13049653; doi:10.1016/j.dialog.2026.100296)
Supplement: Supplementary file 1 — Supplementary material MMAT table [file mmc1.docx]

| f | Authors (Year) | Study Design | Quality | Relevance | Notes |
| --- | --- | --- | --- | --- | --- |
| 1 | Dean SV et al. (2014) | Narrative review | Medium | High | Good overview on maternal care continuum |
| 2 | Hill B et al. (2020) | Network position paper | Medium | High | Global priorities on maternal obesity, relevant scope |
| 3 | Blair A et al. (2022) | Scoping review | High | High | Focus on maternity care for women with disabilities |
| 4 | Jackson-Best F et al. (2018) | Systematic review | High | Medium | Stigma/intersectionality, broad but relevant |
| 5 | Nguyen TV (Doctoral thesis) | Qualitative | Medium | High | Direct lived experience of women with disabilities |
| 6 | Gleason JL et al. (2021) | Quantitative cohort | High | High | Strong data on maternal risks in disabled women |
| 7 | Ngwena CG (2018) | Legal analysis | Medium | Medium | Focus on reproductive autonomy, relevant framework |
| 8 | McCauley H et al. (2022) | Systematic review | High | Medium | Postnatal care focus, useful but broader than disabilities |
| 9 | Hughes RB et al. (2022) | Qualitative | Medium | High | Health info-seeking by disabled women, relevant |
| 10 | Ganguly S, Bajpai K (1994) | Political analysis | Low | Low | Kashmir crisis, less relevant to health/disability |
| 11 | Ilyas M (2024) | Social science paper | Medium | Medium | Peace education in Kashmir, indirectly relevant |
| 12 | Khanday SA (2024) | Review | Medium | High | Public health in Kashmir, relevant local context |
| 13 | Farooq T & Manzoor S (2022) | Qualitative | Medium | High | Parenting & disability lived experience in Kashmir |
| 14 | Khan HN & Digal G (2023) | Mixed methods | Medium | High | Women’s health issues in border Kashmir villages |
| 15 | Ahmad S et al. (2024) | Qualitative | Medium | High | Derogatory labels towards disabled persons in Kashmir |
| 16 | Begley C et al. (2009) | Qualitative review | High | High | Barriers for disabled women accessing maternal services |
| 17 | Wani RT et al. (2019) | Cross-sectional | Medium | Medium | Family planning knowledge among healthcare workers in Kashmir |
| 18 | Farooqi N & Ali M (2023) | Literature review | Medium | Medium | Disability paradigms overview |
| 19 | Moher D et al. (2009) | Methodology paper | High | Medium | PRISMA statement, relevant for review methodology |
| 20 | Sharma R et al. (2023) | Mixed methods | Medium | High | User-friendliness of reproductive services for disabled |
| 21 | Deierlein AL et al. (2021) | Systematic review | High | High | Pregnancy outcomes in disabled women |
| 22 | Tak JA (2022) | Journalistic article | Low | Medium | Narrative on women with disabilities in Kashmir |
| 23 | Pathak M (2022) | Journalistic article | Low | Medium | Disability situation in Kashmir, descriptive |
| 24 | Addlakha R et al. (2017) | Review | Medium | High | Disability and sexual/reproductive rights |
| 25 | Becker H et al. (1997) | Qualitative study | Medium | High | Reproductive health experiences of disabled women |
| 26 | Bashir N & Dar JA (2023) | Review | Medium | High | Maternal health challenges and remedies for disabled women |
| 27 | Matin BK et al. (2021) | Systematic review | High | High | Healthcare access barriers for women with disabilities |
| 28 | O'Connor-Terry C & Harris J (2022) | Qualitative | Medium | High | Pregnancy decision-making by disabled women |
| 29 | Hajira S (2023) | Policy review | Medium | Medium | Disability rights in India, broader policy context |
| 30 | Farooq A et al. | Report/Review | Medium | High | Health system challenges in Kashmir |
| 31 | Nguyen TV et al. (2019) | Qualitative review | High | High | Maternal healthcare challenges in low/mid income countries |
| 32 | Smith E et al. (2004) | Qualitative | Medium | High | Barriers to reproductive health for disabled women |
| 33 | Potvin LA et al. (2016) | Qualitative | Medium | Medium | Social support for women with intellectual disabilities |
| 34 | Lawler D et al. (2013) | Systematic review | High | High | Access to maternity services for disabled women |
| 35 | Jamieson R et al. (2016) | Qualitative | Medium | Medium | Supported decision-making for mothers with disabilities |
| 36 | Payne DA et al. (2014) | Qualitative | Medium | Medium | Care for disabled mothers during pregnancy and birth |
| 37 | Carvalho CFS & Brito RS (2016) | Qualitative | Medium | Medium | Support networks in pregnancy for women with disabilities |
| 38 | Powell RM et al. (2017) | Qualitative | Medium | High | Family attitudes towards pregnancy among disabled women |
| 39 | Holmes TH & Rahe RH (1967) | Psychometric scale | High | Low | Stress scale, not directly related to disability or maternity |
| 40 | Höglund B & Larsson M (2013) | Qualitative | Medium | High | Motherhood experiences of women with intellectual disabilities |
| 41 | Lantz PM et al. (2005) | Survey | Medium | Medium | Role of doulas in childbirth, general maternal support |
| 42 | Tarasoff LA (2015) | Literature review | High | High | Perinatal experiences of women with physical disabilities |
| 43 | Guerin BM et al. (2017) | Qualitative | Medium | High | Recommendations to improve maternity support for disabled |
| 44 | Mitra M et al. (2016) | Mixed methods | High | High | Unmet needs of disabled pregnant women |
| 45 | Shikako K et al. (2023) | Policy analysis | Medium | Medium | Government responses for disabled during COVID-19 |
| 46 | US Dept. of Justice (2020) | Policy guidance | High | Medium | Access to medical care for mobility disabled persons |
| 47 | Bhat FA et al. (2014) | Cross-sectional | Medium | High | Gender and health services in Jammu and Kashmir |
| 48 | Smeltzer SC et al. (2016) | Qualitative | Medium | High | Perinatal experiences of women with disabilities |
| 49 | Casebolt MT et al. (2023) | Secondary data analysis | Medium | High | Maternity healthcare use among disabled in Rajasthan |
| 50 | Mitra M et al. (2017) | Qualitative | Medium | High | Healthcare practitioner perspectives on disabled maternity care |
| 51 | Homeyard C et al. (2016) | Systematic review | High | High | Antenatal care for women with intellectual disabilities |
| 52 | Saeed G et al. (2022) | Qualitative | Medium | High | Communication barriers in perinatal care for disabled |
| 53 | Mason MG (2012) | Book | Medium | Medium | Lessons from mothers with disabilities |
| 54 | Zahoor N et al. (2020) | Qualitative | Medium | Medium | Health seeking behavior of tribal pregnant women Kashmir |
| 55 | Fritsch K (2017) | Book chapter | Medium | Medium | Disabled parenting and neoliberal affects |
| 56 | Kayama M et al. (2021) | Qualitative | Medium | Medium | Disability experiences in Indian socio-cultural context |
| 57 | Ven C et al. (2025) | Scoping review | High | High | Factors influencing disability-inclusive maternity care |
| 58 | Smith DD & Tyler NC (2011) | Review | Medium | Low | Inclusive education, less directly related |
| 59 | Sukhera J (2019) | Review | Medium | Low | Empathy training in healthcare, indirectly related |
| 60 | Pinto-Coelho L et al. (2023) | Experimental study | Medium | Low | VR for empathy training, less related |
| 61 | Tarasoff LA (2017) | Qualitative review | High | High | Barriers to perinatal care for women with physical disabilities |
| 62 | Piepzna-Samarasinha LL | Book | High | High | Disability justice, collective care, intersectionality, and community access |

Table 2. Quality appraisal of included studies using the Mixed Methods Appraisal Tool (MMAT)
